# Frequency and risk factors for postoperative aneurysm residual after microsurgical clipping

**DOI:** 10.1007/s00701-020-04639-5

**Published:** 2020-11-20

**Authors:** Kathrin Obermueller, Isabel Hostettler, Arthur Wagner, Tobias Boeckh-Behrens, Claus Zimmer, Jens Gempt, Bernhard Meyer, Maria Wostrack

**Affiliations:** 1 Department of Neurosurgery, Klinikum rechts der Isar, Technical University Munich, Ismaninger Straße 22, 81675 Munich, Germany; 2 Department of Neuroradiology, Klinikum rechts der Isar, Technical University Munich, Ismaninger Straße 22, 81675 Munich, Germany

**Keywords:** Intracranial aneurysm, Aneurysm clipping, Aneurysm residual, Aneurysm remnant, Aneurysm regrowth

## Abstract

**Objective:**

Aneurysm residuals after clipping are a well-known problem, but the course of aneurysm remnants in follow-up is not well studied. No standards or follow-up guidelines exist for treatment of aneurysm remnants. The aim of this study was to evaluate the risk factors for postoperative aneurysm remnants and their changes during follow-up.

**Methods:**

We performed a retrospective analysis of 666 aneurysms treated via clipping in our hospital from 2006 to 2016. Postoperative and follow-up angiographic data were analyzed for aneurysm remnants and regrowth. Clinical parameters and aneurysm-specific characteristics were correlated with radiological results.

**Results:**

The frequency of aneurysm residuals was 12% (78/666). Aneurysms located in the middle cerebral artery (*p* = 0.02) showed a significantly lower risk for incomplete aneurysm occlusion. Larger aneurysms with a diameter of 11–25 mm (*p* = 0.005) showed a significantly higher risk for incomplete aneurysm occlusion. Five patients underwent re-clipping during the same hospital stay. Remnants were stratified based on morphological characteristics into “dog ears” (*n* = 60) and “broad based” (*n* = 13). The majority of the “dog ears” stayed stable, decreased in size, or vanished during follow-up. Broad-based remnants showed a higher risk of regrowth.

**Conclusions:**

A middle cerebral artery location seems to lower the risk for the incomplete clip occlusion of an aneurysm. Greater aneurysm size (11–25 mm) is associated with a postoperative aneurysm remnant. The majority of “dog-ear” remnants appear to remain stable during follow-up. In these cases, unnecessarily frequent angiographic checks could be avoided. By contrast, broad-based residuals show a higher risk of regrowth that requires close imaging controls if retreatment cannot be performed immediately.

## Introduction

The frequency of incomplete aneurysm occlusion after surgical clipping varies from 2–49% in different surgical series [1–5, 7, 9, 12, 13, 15, 20, 24, 25, 27, 28, 31, 32]. The risk of rebleeding after aneurysm clipping is estimated at 1.3% and is associated with the size of the residual rest [19]. However clear criteria for retreatment and for follow-up controls of aneurysm residuals have not yet been strictly defined. Possible risk factors and potential follow-up dynamics are essential for treatment decisions, as well as for patient consultation.

Postoperative angiography is an accepted standard in most neurosurgical departments, as it reveals residual filling of aneurysms or other complications, like major vessel occlusion [13, 20, 27, 28]. In patients with completely clipped aneurysms, further angiographic controls for the treated aneurysms are considered unnecessary. However, no standards have been established for follow-up (FU) in cases with residual aneurysms after clipping. Currently, aneurysm remnants are categorized according to their morphological characteristics. David et al. and Raymond et al. classified aneurysm remnants into 2 categories: “dog-ear” and “broad-based” residuals. “Dog ears” consist of a small neck remnant between the parent vessel and the base of the clip, whereas the reconstructed parent vessel of “broad-based” remnants contains part of the aneurysm wall [9, 29]. These differences in morphology suggest different risks for regrowth and subsequent rebleeding. Accordingly, the follow-up periods and the decision for surgery should be made based on the estimated risk of rupture.

The aim of this study was to determine the frequency and risk factors of residual aneurysm filling after aneurysm clipping and to estimate the risk of regrowth of remnants in our surgical series.

## Methods

We performed a retrospective, single-center data analysis of patients treated for intracranial aneurysm via clipping. The analyzed data were collected from 2006 until 2016.

### Patient inclusion/exclusion criteria

Based on the operational key for aneurysm clipping, the patient data were consecutively entered into the database. We included patients with clipping for incidental aneurysms and patients who were clipped for ruptured aneurysms. Patients who received surgical treatment other than clipping (e.g., wrapping or trapping and bypass surgery) or, who did not undergo postoperative digital subtraction angiography (DSA) were excluded from the final analysis. Patients who underwent aneurysm clipping in the context of other pathologies (for example, arteriovenous malformation, or mycotic aneurysms) were also excluded from the analysis. The local ethics committees approved the study protocol (nb. 5020/11)

### Interventions

Every patient received a four-vessel catheter DSA before and a target vessel DSA within the first 24 h after surgery. The decision for the treatment modality was based on interdisciplinary discussion between the neuroradiologist and neurosurgeon. All surgeries were performed by three experienced vascular neurosurgeons. For all incidental aneurysms, and partly for ruptured aneurysms, patients underwent intraoperative neuromonitoring comprising trans-cranially recorded motor evoked potentials. Indocyanine green (ICG) angiography was routinely conducted. If any aneurysm residual or vessel occlusion was suspected in postoperative angiography, the findings were discussed immediately to provide the possibility of a prompt clip adjustment. Indications for FU angiography in cases of residual filling without an indication for clip repositioning were discussed interdisciplinarily and were performed depending on the size, shape, and general condition of the patient.

### Outcomes

The following clinical, radiological, and epidemiological data were recorded for each patient: age, sex, preoperative neurological condition, Hunt and Hess grade, Fisher grade, aneurysm location and size, number of aneurysms, number of operations, postoperative neurological condition according to the modified Rankin Scale, size of aneurysm remnant, size of aneurysm remnant in FU angiography, and neurological status during FU.

For the present research, postoperative DSA results were screened for aneurysm remnants. An aneurysm remnant was defined as an inflow of contrast medium with a minimum size of 1 mm and visible on at least 2 projections of angiography in the area of a previously described aneurysm. Follow-up examinations were reviewed for growth of the aneurysm remnant, defined as any new inflow of contrast medium (compared to postoperative angiography) that caused an enlargement of the residual. Changes in flow patterns or thrombosis of the remnant were also evaluated.

Aneurysm remnants were divided according to classification of David et al. and Raymond et al. into “dog-ear” remnants with a small residual between the parent vessel and the base of the clip and “broad-based” remnants, in which a larger residual was still filling [9, 29]. The “dog-ear remnant” group was scheduled for FU angiography depending on the remnant size, the history of any previous subarachnoid hemorrhage (SAH), and the general condition and age of the patient. Patients with “broad-based remnants” were either re-operated during the same hospital stay or were designated for FU imaging. FU DSA examinations were scheduled depending on the individual risk factors and remnant characteristics regularly within a period of 3 months to 1 year after surgery. The mean FU in aneurysm remnants was 31 months (SD: ± 23.3, range 2.3–93 months).

### Statistical analysis

The study sample was described using means ± standard deviations for the continuous variables, while categorical parameters were depicted using absolute and relative frequencies. Chi-squared tests and binary logistic regression were used to test categorical variables. Continuous variables were tested with a *t* test or Wilcoxon test. A *p* value of 0.05 was considered statistically significant. Analyses were performed utilizing R Studio Version 1.0.4 (R Studio, Boston, USA).

## Results

### Data collection

**Fig. 1:**
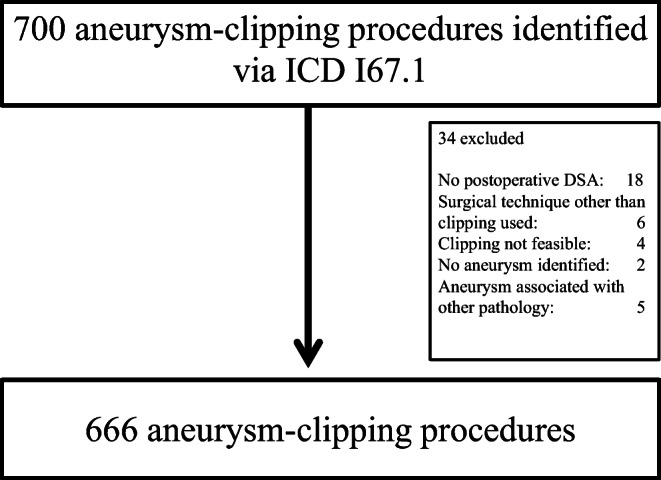
Flowchart of procedure inclusion via ICD code I67.1

In 492 patients, a total of 666 ruptured and unruptured aneurysms were treated between April 2006 and December 2016. The reasons for case exclusion are listed in Fig. 1.

In 18 patients, no postoperative DSA was performed. In this group, 11 cases had suffered a severe SAH and had died soon after aneurysm clipping. In 3 cases, other medical circumstances (malignant disease) were treated immediately and were judged more important than the aneurysm. Complications during the initial angiography, with arterial dissection in 2 and symptomatic arterial emboli in 1 case, led to no postoperative DSA. One patient rejected postoperative DSA after elective clipping.

### Patient data

Patient data and data on the treated aneurysms are shown in Tables 1 and 2.

**Table 1:** Patient data and clinical characteristics (*n* = 492)

Parameter	Value
Age at surgery mean ± SD	55.48 ± 13.1
Gender female *n*, (%)	343 (70)
Acute SAH *n*, (%)	252 (51)
Multiple aneurysms ≥ 2 *n*, (%)	167 (34)

*SAH*, subarachnoid hemorrhage

**Table 2:** Data on treated aneurysms (*n* = 666)

Parameter	Value
Aneurysm location *n*, (%)	
ACA	152 (23)
ICA	140 (21)
MCA	340 (51)
PC	34 (5)
Aneurysm size *n*, (%)	
< 3 mm	136 (20.4)
3–6 mm	306 (46.0)
7–10 mm	129 (19.4)
11–25 mm	59 (8.8)
> 25 mm	4 (0.6)
Residual of coiled aneurysm	32 (4.8)

*ACA*, anterior cerebral artery; *ICA*, interior carotid artery; *MCA*, middle cerebral artery; *PC*, posterior circulation

### Frequency of postoperative aneurysm remnants

Residual filling was found in 78/666 aneurysms (12%). Data on patients with postoperative aneurysm residuals are presented in Table 3.

**Table 3:** Data on aneurysms with residual filling in postoperative DSA (*n* = 78). The percentage is based on the total number of operated patients in the respective subgroup

Parameter	Value
Aneurysm location	*n*, (% of the entire subgroup)
ACA (*n* = 152)	18 (11)
ICA (*n* = 140)	28 (20)
MCA (*n* = 340)	25 (7)
PC (*n* = 34)	8 (24)
Aneurysm size *n*, (%)	
< 3 mm (*n* = 136)	7 (5)
3–6 mm (*n* = 306)	28 (9)
7–10 mm (*n* = 129)	23 (17)
11–25 mm (*n* = 59)	18 (31)
> 25 mm (*n* = 4)	1 (25)
Residual of coiled aneurysm (*n* = 32)	2 (6)

*ACA*, anterior cerebral artery; *ICA*, interior carotid artery; *MCA*, middle cerebral artery; *PC*, posterior circulation

### Risk of aneurysm residuals

Binary logistic regression revealed a higher risk of an aneurysm residual for aneurysms sized 11–25 mm, with an odds ratio of 9.36 (CI 1.96–44.61; *p* = 0.005). A location in the MCA showed a significantly lower risk for aneurysm residuals when compared with other locations, with an odds ratio of 0.31 (CI: 0.12–0.83; *p* = 0.02) (Table 4). Sex, number of aneurysms per patient, and emergency setting of surgery showed no significant association with postoperative aneurysm residuals.

**Table 4:** Factors associated with postoperative aneurysm residuals. The factor positively associated with postoperative aneurysm residuals is aneurysm size 11–25 mm. The location of the “MCA” aneurysm is associated with a reduced risk for postoperative aneurysm residuals. (Binary logistic regression)

Parameter	OR (95%CI)	*p*
Aneurysm location		
ACA	0.67 (0.24–1.84)	0.44
ICA	1.35 (0.51–3.61)	0.55
MCA	0.31 (0.12–0.83)	0.02
Aneurysm size *n* (%)		
< 3 mm	0.98 (0.19–4.99)	0.98
3–6 mm	2.04 (0.46–9.12)	0.35
7–10 mm	4.23 (0.95–20.60)	0.06
11–25 mm	9.36 (1.96–44.61)	0.005
> 25 mm	4.46 (0.37–66.74)	0.28
Surgery in acute phase	0.67 (0.38–1.16)	0.16

*ACA*, anterior cerebral artery; *ICA*, interior carotid artery; *MCA*, middle cerebral artery

### Follow-up of aneurysm remnants

**Fig. 2:**
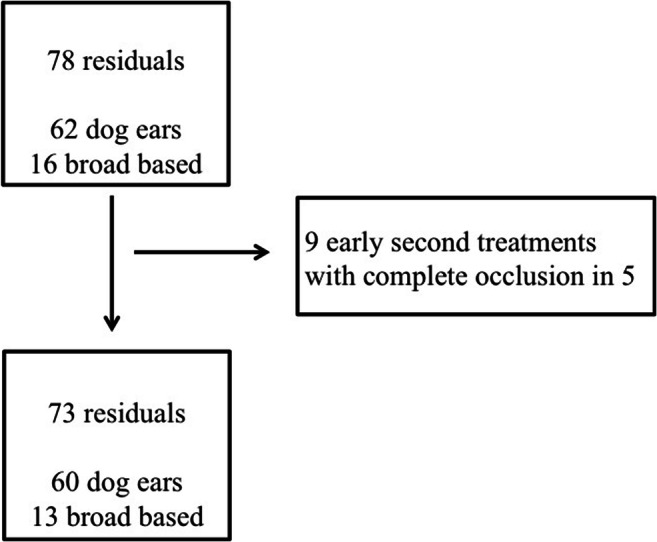
Flowchart of aneurysm residuals

Figure 2 shows the classification and early treatment of aneurysm remnants. FU was available for 33/60 (55%) aneurysms in the dog-ear group and for 7/13 (54%) in the group of broad-based residuals. The mean FU was 31 months (SD: ± 23.3, range 2.3–93 months).

The reasons for missing FU differed. In 12 cases, FU angiography was recommended but the patient did not attend the FU examination. In 7 cases, the patients were in poor neurological condition and FU angiography was recommended only if the patients improved during their postoperative courses. These were all patients who had suffered a severe SAH. In 4 dog-ear cases, FU angiography was not recommended because of the small size of the aneurysm remnant. In 9 cases, a FU recommendation was not specified at discharge.

Follow-up of dog-ear remnants and broad-based remnants

Table 5 shows the course of dog-ear residuals during FU. The major part of the dog-ear residuals stayed stable, decreased in size, or closed during FU (94%). An increase in size was recorded in two cases.

**Table 5:** Behavior of dog-ear residuals in follow-up (*n* = 33)

	Stable size in FU	Decreased size in FU	Closed in FU	Increased in size
Number of aneurysm residuals *n*, (%)	22 (67)	3 (9)	6(18)	2 (6)

**Fig. 3:**
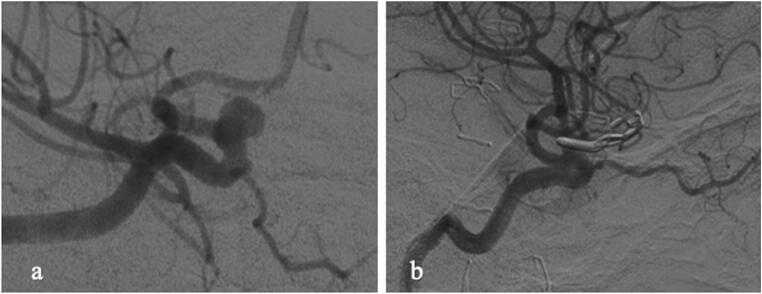
**a** Preoperative DSA demonstrating an incidental paraophthalmic ICA aneurysm which was scheduled for clipping; **b** postoperative DSA reveals small dog-ear residual. This patient was scheduled for follow-up DSA, which showed a stable result

**Fig. 4:**
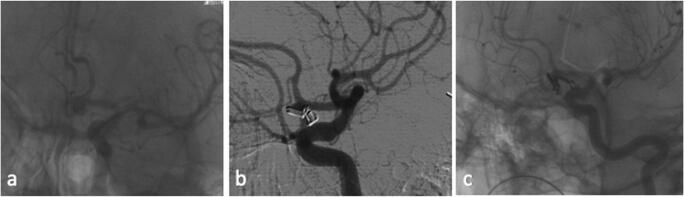
**a** Preoperative DSA of an Acoma aneurysm in a patient with SAH H&H II; **b** postoperative DSA 1 showed a broad-based aneurysm residual and the patient underwent revision surgery on the same day; **c** postoperative DSA 2 demonstrates now complete occlusion of the residual, which was achieved by placement of further clips

During FU, two of the broad-based aneurysms increased in size, one decreased, and none closed spontaneously. The number of broad-based aneurysms in FU was small (*n* = 7) (Table 6). Figures 3 and 4 show typical DSA findings of a dog ear and a broad-based aneurysm residual.

**Table 6:** Behavior of broad-based residuals in follow-up (*n* = 7)

	Stable size in FU	Decreased size in FU	Increased in size
Number of aneurysm residuals *n*, (%)	4 (60)	1 (14)	2 (28)

### Treatment of aneurysm residuals during follow-up

All aneurysm remnants with increasing size were treated (2 in the dog-ear group, 2 in the broad-based group). The 2 progressive dog-ear residuals were treated 1 year after clipping by coiling and stent-assisted coiling. The progressive broad-based residuals were treated via clipping in one case at 6 months after the first surgery and by coiling in the other case at 1 year after surgery.

Two broad-based aneurysm residuals were treated irrespective of the residual growth during the FU. In 1 case, the broad-based residual of a paraophthalmic aneurysm was treated with a flow diverter 6 months postoperatively. In another case, a paraophthalmic ICA aneurysm was clipped and postoperative angiography revealed a small remnant. Early second surgery was performed, but a postoperative DSA still showed an aneurysm residual. One year later, the aneurysm residual was treated with stent-assisted coiling and showed no further growth during FU.

## Discussion

In the current study, we analyzed the risk factors for aneurysm remnants after microsurgical clipping and the course of aneurysm remnants in FU. Postoperative angiography revealed a rate of 12% for residual aneurysm filling in clipped aneurysms in our series. These data are consistent with other published studies, as the frequency of residual filling of aneurysms after clipping is reported to range from 2.3 to 19.3%. One series from Korea revealed an incidence of residual filling of even 49% when controlled with 3D angiography and defining a remnant size from 1 mm onward [1–5, 7, 9, 12, 13, 15, 20, 24, 25, 27, 28, 31, 32]. Our series confirms the need for early postoperative angiography to certify the success of the surgery and, in the case of remnants, to ensure that FU or further therapy can be scheduled appropriately. The necessity for postoperative angiography has been discussed previously [8, 10, 12], and this procedure remains the absolute standard for control after aneurysm surgery.

### Risk factors for incomplete aneurysm occlusion

A greater aneurysm size (11–25 mm) was associated with incomplete surgical occlusion. Jabbarli et al. described similar results for aneurysms sized > 12 mm and an association with a higher risk for a clip remnant [18].

The location of the aneurysm is considered important for the occurrence of an aneurysm remnant, as the risk of aneurysm remnants seem to be greater for paraophthalmic, anterior communicating artery, and basilar artery aneurysms [18, 27, 32]. Our series revealed a lower risk for aneurysm residuals following clipping of MCA aneurysms, in agreement with previously described findings and reflecting the technically easier accessibility as compared to paraophthalmic and posterior circulation aneurysms [21, 28].

Direct intraoperative catheter angiography within a hybrid operating room is described as potentially beneficial for the occlusion rates of the aneurysms [22, 26]. However, in our series, only 5 patients underwent early clip revision surgery. This was primarily due to the technically difficult configuration of the aneurysm; therefore, it could not be avoided with the use of intraoperative DSA.

Angiographic and clinical follow-up of aneurysm remnants

In our series, the major part of dog-ear residuals remained stable, decreased in size, or vanished during the FU. Therefore, foregoing angiographic controls might be justified, particularly in patients without risk factors such as a previous history of SAH, hereditary or de novo aneurysms, hypertonus, or smoking. A younger age should also be regarded as a potential risk factor because of the previously reported increased risk of regrowth of aneurysm residuals in patients under 45 years old [18].

In 2004, Akyüz et al. performed a long-term angiographic FU (median 46.6 months) of 166 clipped aneurysms, with 7 residuals (4.2%), using the Sindou residual grading system. They reported 5 small neck residuals and 2 with a broader residual. One case of regrowth was recorded in a small remnant and led to reoperation, but no cases of regrowth occurred among the larger remnants. These surgeons also had one case of spontaneous thrombosis of a small remnant but experienced no cases of rebleeding during FU. They concluded that small aneurysm remnants may stay stable [3].

The FU of broad-based aneurysms in our series was incomplete, but it revealed a high rate of regrowth (28%). This was also observed by David et al., who noted aneurysm regrowth in 3 out of 4 cases [9]. Only one broad-based residual decreased in size during FU, but it did not close completely. Broad-based residuals seem to have a high risk of regrowth and should be scheduled for FU, as the need for retreatment seems to occur frequently. Furthermore, the CARAT study revealed a size-dependent risk of rebleeding in clipped and coiled aneurysms [19].

In treated aneurysms after SAH, the risk of rehemorrhage has been estimated as 0.11–0.21% in the first year for coiled aneurysms and 0.0–0.03% for clipped aneurysms [10, 19]. In our series, no case of rebleeding occurred in the incompletely clipped aneurysms within the FU of a maximum of 93 months. David et al. estimated the risk of rebleeding in aneurysms with postoperative remnants as 1.5% per year [9]. A series of 715 surgically treated patients reported by Feuerberg et al. revealed a risk of rebleeding in aneurysm residuals of 0.38–0.79% per year, leading to a discussion of whether this risk of rebleeding might justify the risk of reoperation [15]. Rauzzino et al. determined a relatively high risk of rebleeding in aneurysm remnants. Surgery in 312 aneurysms led to a remnant rate of 4.2% (*n* = 13). Only 4 of these remnants were not treated immediately after initial surgery. All 4 of these remaining aneurysm remnants became symptomatic within 2 years: 3 with a new SAH and 1 because of a local mass effect [28]. Configuration of the remnants was not further specified in this study.

Furthermore, radiation exposure, periprocedural complications, and costs should also be factored into the decision for invasive angiographic controls. The development of less invasive but adequate imaging procedures with lower radiation exposure for the patient, such as metal artifact reduced MRI, would be desirable. However, even the most recent, newly developed MRI sequences are not able to suppress the artifacts so strongly to be able to determine the fine differences in size or configuration of the neck remnant [16]. The MRI is nowadays much more useful in checking untreated aneurysms or in excluding de novo formations in predisposed patients.

CT angiography (CT-A) is less invasive and has been compared to classical DSA in different studies. The sensitivity for detection of postoperative aneurysm remnants varied between 50–100% comparing CT-A to DSA [6, 11, 14, 23, 30, 33, 34]. The precision of the CT-A strongly decreases with the usage of multiple clips, in cases with posterior circulation aneurysms and in small remnants.

Sagara et al. came to the conclusion that the usage of multiple clips is an indication for 3D DSA [30]. Bharatha et al. reported a sensitivity of 88% for the detection of aneurysm remnants after clipping when comparing DSA to CT-A, whereas the sensitivity for a small neck remnant (mean size 1 mm) was only 20% [6].

Dundar et al. compared traditional DSA to subtraction CT-A and came to the conclusion that DSA remains the gold standard as residuals < 3 mm are not reliably examined with CT-A [14].

In our opinion and according to previous publications, CT-A might be suitable, for long-term follow-up controls when DSA controls were initially stable and CT-A quality is good [17].

### Study limitations

One limitation of our study is its retrospective data collection. Furthermore, FU is not available for almost half the patients with incompletely occluded aneurysms, either due to the poor clinical condition of certain SAH patients or due to the estimated low-risk factors based on patient’s postoperative angiography and medical history. This may obscure the true data concerning growth dynamics of incompletely occluded aneurysms.

## Conclusions

MCA aneurysms appear to have a lower risk for postoperative aneurysm residuals than is observed for an aneurysm in other locations. A large part of dog-ear residuals appear to remain stable, decrease in size, or close during FU. Management of aneurysm remnants should consider individual risk factors and the remnant configuration.

## Abbreviations

ACA: Anterior cerebral artery
DSA: Digital subtraction angiography
Fig.: Figure
FU: Follow-up
H&H: Hunt and Hess grade
ICA: Internal carotid artery
ICD: International Statistical Classification of Diseases and Related Health Problems
ICG angiography: Indocyanine green angiography
MCA: Middle cerebral artery
PC: Posterior circulation
SAH: Subarachnoid hemorrhage
SD: Standard deviation
Tab.: Table

## References

[1] Acevedo JC, Turjman F, Sindou M (1997). Postoperative arteriography in surgery for intracranial aneurysm. Prospective study in a consecutive series of 267 operated aneurysms. Neuro-Chirurgie.

[2] Ahn SS, Kim YD (2010). Three-dimensional digital subtraction angiographic evaluation of aneurysm remnants after clip placement. J Korean Neurosurg Soc.

[3] Akyuz M, Tuncer R, Yilmaz S, Sindel T (2004). Angiographic follow-up after surgical treatment of intracranial aneurysms. Acta Neurochir.

[4] Allcock JM, Drake CG (1963). Postoperative angiography in cases of ruptured intracranial aneurysm. J Neurosurg.

[5] Asgari S, Doerfler A, Wanke I, Schoch B, Forsting M, Stolke D (2002). Complementary management of partially occluded aneurysms by using surgical or endovascular therapy. J Neurosurg.

[6] Bharatha A, Yeung R, Durant D, Fox AJ, Aviv RI, Howard P, Thompson AL, Bartlett ES, Symons SP (2010). Comparison of computed tomography angiography with digital subtraction angiography in the assessment of clipped intracranial aneurysms. J Comput Assist Tomogr.

[7] Choi SW, Ahn JS, Park JC, Kwon do H, Kwun BD, Kim CJ (2012). Surgical treatment of unruptured intracranial middle cerebral artery aneurysms: angiographic and clinical outcomes in 143 aneurysms. J Cerebrovasc Endovasc Neurosurg.

[8] Cronk K, Spetzler RF (2010). Commentary for recurrent intracranial aneurysms after successful neck clipping. World Neurosurg.

[9] David CA, Vishteh AG, Spetzler RF, Lemole M, Lawton MT, Partovi S (1999). Late angiographic follow-up review of surgically treated aneurysms. J Neurosurg.

[10] de Sousa AA (2010). Follow-up of cerebral aneurysms after neck clipping. World Neurosurg.

[11] Dehdashti AR, Binaghi S, Uske A, Regli L (2006). Comparison of multislice computerized tomography angiography and digital subtraction angiography in the postoperative evaluation of patients with clipped aneurysms. J Neurosurg.

[12] Drake CG, Allcock JM (1973). Postoperative angiography and the “slipped” clip. J Neurosurg.

[13] Drake CG, Friedman AH, Peerless SJ (1984). Failed aneurysm surgery. Reoperation in 115 cases. J Neurosurg.

[14] Dundar TT, Aralasmak A, Kitiş S, Yılmaz FT, Abdallah A (2019). Comparison of subtracted computed tomography from computed tomography perfusion and digital subtraction angiography in residue evaluation of treated intracranial aneurysms. World Neurosurg.

[15] Feuerberg I, Lindquist C, Lindqvist M, Steiner L (1987). Natural history of postoperative aneurysm rests. J Neurosurg.

[16] Friedrich B, Wostrack M, Ringel F, Ryang YM, Forschler A, Waldt S, Zimmer C, Nittka M, Preibisch C (2016). Novel metal artifact reduction techniques with use of slice-encoding metal artifact correction and view-angle tilting mr imaging for improved visualization of brain tissue near intracranial aneurysm clips. Clin Neuroradiol.

[17] Gölitz P, Struffert T, Ganslandt O, Saake M, Lücking H, Rösch J, Knossalla F, Doerfler A (2012). Optimized angiographic computed tomography with intravenous contrast injection: an alternative to conventional angiography in the follow-up of clipped aneurysms?. J Neurosurg.

[18] Jabbarli R, Pierscianek D, Wrede K, Dammann P, Schlamann M, Forsting M, Muller O, Sure U (2016) Aneurysm remnant after clipping: the risks and consequences. J Neurosurg 125(5):1249–1255. 10.3171/2015.10.jns151536 10.3171/2015.10.JNS151536 26871206

[19] Johnston SC, Dowd CF, Higashida RT, Lawton MT, Duckwiler GR, Gress DR, Investigators C (2008). Predictors of rehemorrhage after treatment of ruptured intracranial aneurysms: the Cerebral Aneurysm Rerupture After Treatment (CARAT) study. Stroke.

[20] Kang HS, Han MH, Kwon BJ, Jung SI, Oh CW, Han DH, Chang KH (2004). Postoperative 3D angiography in intracranial aneurysms. AJNR Am J Neuroradiol.

[21] Kivisaari RP, Porras M, Ohman J, Siironen J, Ishii K, Hernesniemi J (2004). Routine cerebral angiography after surgery for saccular aneurysms: is it worth it?. Neurosurgery.

[22] Klopfenstein JD, Spetzler RF, Kim LJ, Feiz-Erfan I, Han PP, Zabramski JM, Porter RW, Albuquerque FC, McDougall CG, Fiorella DJ (2004). Comparison of routine and selective use of intraoperative angiography during aneurysm surgery: a prospective assessment. J Neurosurg.

[23] Kunert P, Prokopienko M, Gola M, Dziedzic T, Jaworski M, Marchel A (2013). Assessment of long-term results of intracranial aneurysm clipping by means of computed tomography angiography. Neurol Neurochir Pol.

[24] Le Roux PD, Elliott JP, Eskridge JM, Cohen W, Winn HR (1998). Risks and benefits of diagnostic angiography after aneurysm surgery: a retrospective analysis of 597 studies. Neurosurgery.

[25] Macdonald RL, Wallace MC, Kestle JR (1993). Role of angiography following aneurysm surgery. J Neurosurg.

[26] Marbacher S, Mendelowitsch I, Gruter BE, Diepers M, Remonda L, Fandino J (2018) Comparison of 3D intraoperative digital subtraction angiography and intraoperative indocyanine green video angiography during intracranial aneurysm surgery. J Neurosurg 131(1):64–71. 10.3171/2018.1.jns172253 10.3171/2018.1.JNS172253 30004279

[27] Meyer B, Urbach H, Schaller C, Baslam M, Nordblom J, Schramm J (2004). Immediate postoperative angiography after aneurysm clipping--implications for quality control and guidance of further management. Zentralbl Neurochir.

[28] Rauzzino MJ, Quinn CM, Fisher WS (1998). Angiography after aneurysm surgery: indications for “selective” angiography. Surg Neurol.

[29] Raymond J, Roy D, Bojanowski M, Moumdjian R, L’Espérance G (1997). Endovascular treatment of acutely ruptured and unruptured aneurysms of the basilar bifurcation. J Neurosurg.

[30] Sagara Y, Kiyosue H, Hori Y, Sainoo M, Nagatomi H, Mori H (2005). Limitations of three-dimensional reconstructed computerized tomography angiography after clip placement for intracranial aneurysms. J Neurosurg.

[31] Sato S, Suzuki J (1971). Prognosis in cases of intracranial aneurysm after incomplete direct operations. Acta Neurochir.

[32] Sindou M, Acevedo JC, Turjman F (1998). Aneurysmal remnants after microsurgical clipping: classification and results from a prospective angiographic study (in a consecutive series of 305 operated intracranial aneurysms). Acta Neurochir.

[33] Teksam M, McKinney A, Casey S, Asis M, Kieffer S, Truwit CL (2004). Multi-section CT angiography for detection of cerebral aneurysms. AJNR Am J Neuroradiol.

[34] van Loon JJ, Yousry TA, Fink U, Seelos KC, Reulen HJ, Steiger HJ (1997). Postoperative spiral computed tomography and magnetic resonance angiography after aneurysm clipping with titanium clips. Neurosurgery.

